# Vibration Signal Analysis Based on Spherical Error Compensation

**DOI:** 10.3389/fbioe.2022.950580

**Published:** 2022-08-19

**Authors:** Shan Wei

**Affiliations:** Industry Design Department, Xin Xiang Universtiy, Xinxiang, China

**Keywords:** vibrating screen, vibration excitation force, simulation, path of particle, spherical error compensation

## Abstract

A vibrating screen is important equipment in industrial production. According to the principle of bionics, a vibrating screen can be divided into a linear vibrating screen, elliptical vibrating screen, ball vibrating screen, and banana vibrating screen. There are also great problems with the use of a vibrating screen. The vibrating screen works due to the vibration excitation force generated by vibration. This work studies the motion trajectory of a vibrating screen by taking the vibrating screen with line motion trajectory as the research object. In this study, the vibration information is detected by an intelligent sensor, and the signal is filtered by an intelligent algorithm. Then, the spherical error compensation is used to improve the calculation accuracy, and the least square method is used to evaluate the error. Finally, the accurate vibration trajectory of the vibrating screen is obtained. The acquisition of a vibration track can provide the working efficiency and safety performance of the vibrating screen, and has social and economic benefits.

## Introduction

At present, the vibrating screen at home and abroad has the characteristics of high efficiency and high reliability. The working performance of the vibrating screen has been continuously optimized. With the development of the economy, some equal energy vibrating screens such as small vibrating screen and artificial vibrating screen cannot meet people’s needs in life and industry. The development of vibrating screens in China tends to be large-scale and intelligent.

1) Because a large-scale vibrating screen is complex in structural stiffness and strength, and its screen surface mesh is also difficult to manufacture, the service life of the large-scale vibrating screen is relatively low due to structural problems, so it is difficult to manufacture. Although the manufacturing process of a large vibrating screen is cumbersome, especially in terms of its stiffness and strength, the advantages of the large vibrating screen in work cannot be achieved by a small vibrating screen. For example, in the screening stage, the screen surface ratio of a large vibrating screen is large, so the screening efficiency is relatively high (Guo and Kuang, 2009; Wang, 2010; Zhang et al., 2013).
2) The intelligent vibrating screen is also increasingly favored by many enterprises and factories. At present, the vibrating screen is connected with computer software to realize the intelligent vibrating screen (Hou et al., 2003; Kuang, 2009; Du and Chen, 2010). At present, advanced enterprises and factories at home and abroad use the most advanced, reliable, and practical monitoring instruments, use the optimization method of establishing a mathematical model, and conduct timely monitoring and diagnosis of the working and fault state of the elliptical vibrating screen through accurate computer calculation, so as to liberate the vibrating screen from simple mechanical operations. The serialization of a vibrating screen can enable its parameters to be debugged during its work so that it can work within a normal working range, which can not only improve its working efficiency but also prolong its working life (Duan and Guo, 2009; Wang, 2010; Peng, 2012; Zhang and Zhu, 2012). With the development of intelligence, the machine can effectively replace artificial dullness in hearing, vision, and touch, and make use of accurate mathematical models in the process of machine work and high-speed computer programs to achieve rapid real-time detection and control of the machine. Spherical error compensation will be introduced to improve trajectory accuracy.

The development trend at home and abroad should be toward large-scale standardization and serialization, which not only greatly improves the working efficiency of the vibrating screen but also makes the vibrating screen more widely used, and the economic value and use value are more comprehensively applied (Hou et al., 2003; Jiao et al., 2006).

## Composition and Working Principle of a Monitoring System

### Composition and Working Principle of a Hardware System

The monitoring system used in this study is shown in Table 1:

**TABLE 1:** Hardware of the monitoring system.

Component name	Piezoelectric gravity sensor	Three-axis output acceleration sensor	24 V switching power supply	Data acquisition card	Frequency amplifier (transmitter)	Upper computer	Shielding wire
Number	4	1	1	1	4	1	Several

Special attention should be paid to connecting these components together with shielded wire because the detected signal is relatively weak during detection. If these components are not connected with shielded wire, the detected signal may be disturbed by the surrounding environment and cause distortion to the detected signal.

At present, there are two kinds of gravity sensors in use: one is a dynamic sensor and the other is a dynamic and static sensor (Yan, 2007). Here, the static and dynamic sensor is selected in this design, because the vibrating screen vibrates when working, and the vibration is static and dynamic. The dynamic sensor cannot accurately reflect the dynamic real-time effect, so the static and dynamic sensor should be selected. When selecting the transmitter, the voltage type transmitter should be selected, because the output of the gravity sensor is voltage. If the transmitter selects the current type, the transmitter needs to convert the input voltage of the sensor into current and the output into voltage. The detected signal itself is very weak, through the conversion from voltage to current and then to voltage. It is easy to lose the signal, so the voltage type should be selected when selecting the transmitter (Chen, 2004). Before the test, the hydraulic press shall be used to check each sensor.

The hydraulic press is gradually pressurized, and then the error between the actual pressure and the measured pressure is tested. By debugging the transmitter, the hydraulic press is used to test the error between the actual value and the real value again, and finally, the error of the monitored sensor is minimized to ensure that the accuracy of the sensor will not affect the verification accuracy of the whole experiment. Some results of the verification measurement are shown in Table 2. From the data in the table, we can see that the error of each sensor under load is within the error range, and the error under static load is controlled within 100 kg, which is allowed by industrial production conditions.

**TABLE 2:** Verification of measurement results.

Sensor1 (channel 1)			Sensor 2 (channel 2)			Sensor 3 (channel 3)			Sensor 4 (channel 4)		
Display load	Actual load	Error	Display load	Actual load	Error	Display load	Actual load	Error	Display load	Actual load	Error
221	208	13	506	510	−4	119	110	9	505	502	3
516	503	13	1,038	1,038	0	517	512	5	1,015	1,010	5
1,019	1,009	10	1,545	1,545	0	1,008	1,007	1	1,532	1,526	6
1,533	1,528	5	2,130	2,132	7	1,503	1,516	−13	2045	2045	0
2028	2023	5	2,560	2,555	5	2012	2012	0	2,520	2,518	2
2,536	2,536	0	3,040	3,040	0	2,502	2,503	−1	3,010	3,010	0
3,000	3,000	0	3,560	3,564	−4	3,012	3,007	5	3,502	3,506	−4
3,565	3,565	0	4,000	4,010	−10	3,517	3,510	7	4,040	4,054	−14
4,012	4,007	5	4,507	4,527	−20	4,020	4,014	6	4,500	4,503	−3
4,527	4,522	5	5,020	5,030	−10	4,518	4,512	6	5,018	5,014	4

**FIGURE 1:**
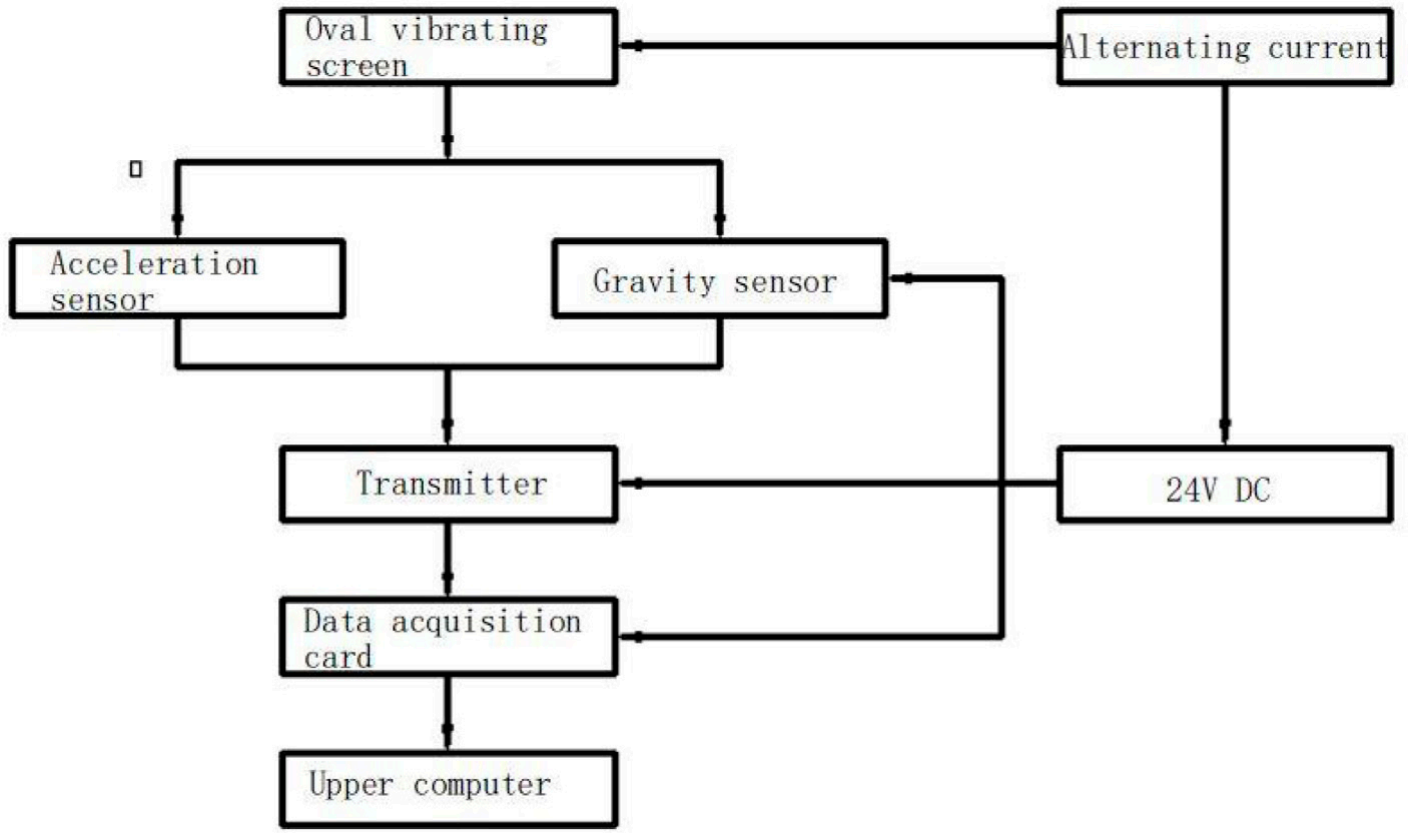
Monitoring system flow.

During measurement, the triaxial voltage signal output acceleration sensor is connected with the frequency amplifier. When the triaxial voltage signal output acceleration sensor is vibrated, its internal piezoelectric plate has a piezoelectric effect. At this time, an alternating charge is generated on its two surfaces. The alternating charge is directly proportional to the force, that is, it is directly proportional to the acceleration of the vibrating screen and passes through the transmitter. The collected signal is transmitted to the data acquisition card in the form of voltage. The data acquisition card converts the voltage into an electrical signal and transmits it to the computer through Ethernet, and then the corresponding software in the computer displays the electrical signal in the form of waves (Xue and Yang, 2002; Zhou, 2004; Zhang et al., 2014). When the vibrating screen vibrates, the piezoelectric gravity sensor placed on the four corners of the vibrating screen receives the impact force from the vibration of the vibrating screen, transmits the collected signal to the computer through the information acquisition card through the frequency amplifier, and the corresponding software in the computer displays the signal collected by the impact force received by the piezoelectric gravity sensor in the form of waves through the operation. The flow chart of the monitoring system of this subject is shown in Figure 1.

### Working Principle of the Software System

The software system plays a key role in the monitoring and signal acquisition of the vibrating screen. The software system collects the running track of the vibrating screen through the impact of the vibrating screen on the gravity sensor and acceleration sensor, and realizes the monitoring of the vibrating screen and the impact on the ground. The software part uses visual builder software to compile the calculation software in the design process and uses an access database to store data and visualize its design calculation. The software part mainly compiles the changes in images and values in the design so that the gravity sensor can quickly and accurately reflect the impact force when it is impacted by the vibrating screen. When the gravity sensor is impacted by the vibration of the vibrating screen, the computer will accurately display the change of the waveform and the change in the weight value. Special attention is paid to that the gravity and pressure here are the same because the pressure and weight have been converted in the program when writing the software, which not only reduces the error of work but can also improve the efficiency of work. The system software platform consists of the following main parts:

1) Data acquisition module: when the gravity sensor and acceleration sensor are vibrated by the vibrating screen, the monitored data are collected through the compiled software system.
2) Waveform display module: the waveform is displayed on the upper computer by the impact force when the gravity sensor and acceleration sensor are vibrated by the vibrating screen.
3) Data analysis module: when the gravity sensor and acceleration sensor are vibrated by the vibrating screen, the impact force displayed on the upper computer is different from the actual load and the displayed fluctuation error (Xue and Yang, 2002).
4) User operation interface: the setting of the user operation interface makes the vibration signal more visual, intuitive, and controllable. Through the waveform display and data display of the vibration signal in the user interface, the staff can quickly and timely see the changes of the vibration signal, to timely adjust the working performance of the vibrating screen in the case of failure of the vibrating screen.

### Data Analysis and Error Compensating

In order to enhance the accuracy, this study puts forward the improved least-squares ellipsoid fitting method. The method is based on the assumption ellipsoid (Madhow and Honig, 1994; Yun et al., 2022). The error compensation coefficient is calculated by the least square method, and the constraint has been solved by the matrix decomposition of the matrix singularity problem to overcome the instability of the algorithm. The approach reduces the amount of calculation time. The software simulation and experiment verify the effectiveness of the algorithm (Schneider, 1979; Jiang et al., 2021a; Huang et al., 2021; Sun et al., 2022).

For a three-axial sensor, its inherent error is mainly characterized by zero error, error sensitivity, orthogonal error, etc. Assuming that the actual output of the sensor is $h_{s}$ , no error exists for the ideal output $h_{t}$ $\left(h_{s}\neq h_{t}\right)$ , and its mathematical model is expressed as follows:

$$h_{s}=k_{d}k_{p}h_{t}+B_{e}=k_{e}h_{t}+B_{e}.$$ (1)

Error matrix $K_{d}$ is a third-order diagonal matrix, which stands for the sensitivity of various shaft sensors. $K_{p}$ stands for non-orthogonality between the axis of the sensors and the soft magnetic material part. Then, a sensor proper reference coordinate system can be established. $K_{p}$ can be presented by a third-order diagonal matrix. $B_{e}$ stands for the sensor’s zero error and hard magnetic materials. Error compensation of sensors equals to determine the error coefficient matrix $K_{e}$ and $B_{e}$ . By the known actual output $h_{s}$ , to solve the ideal output $h_{t}$ , the following equation is used:

$$h_{t}=K_{c}\left(h_{s}+B_{c}\right).$$ (2)

In Eq. 2, $k_{c}=k_{e}^{-1}$ , $B_{c}=-B_{e}$ .

The vector of $h_{t}$ is in the form of

$$\left[\begin{array}{l} h_{x_{t}}\\ h_{y_{t}}\\ h_{z_{t}} \end{array}\right]=\left[\begin{array}{ccc} k_{11} & 0 & 0\\ k_{21} & k_{22} & 0\\ k_{31} & k_{32} & k_{33} \end{array}\right]\left(\left[\begin{array}{c} h_{x_{s}}\\ h_{y_{s}}\\ h_{z_{s}} \end{array}\right]+\left[\begin{array}{c} b_{1}\\ b_{2}\\ b_{3} \end{array}\right]\right).$$ (3)

At a certain moment for a fixed position, assume that the magnetic field strength and the direction are constant, the rotation of the sensor is in a three-dimensional space, and the ideal output data within the space of trajectory are spherical, then

$${\left\|h_{t}\right\|}^{2}=H^{2},$$ (4)

where *H* means the location of the magnetic field intensity. Combining Eq. 2 with Eq. 4, we can get

$$h_{s}^{T}Ah_{s}-2b^{T}Ah_{s}+b^{T}Ab=H^{2}.$$ (5)

where $A=K_{C}^{T}K_{C}$ and $b=-B_{C}$ , based on the assumption that the ellipsoid compensation approach considers the measurements of the actual output trajectory to be an ellipsoid, namely, Eq. 5 said ellipsoid equation of vector. The problem of error compensation of sensors becomes an ellipsoid fitting problem.

Changing Eq. 5 into the general equation of a quadric surface, then we can get

$$\begin{array}{l} F\left(\alpha ,X\right)=a_{1}x^{2}+a_{2}xy+a_{3}y^{2}+a_{4}xz+a_{5}yz+\\ a_{6}z^{2}+a_{7}x+a_{8}y+a_{9}z+a_{10}\\ =\alpha^{T}x=o, \end{array}$$ (6)

$$x={\left[\begin{array}{cccccccccc} x^{2} & xy & y^{2} & xy & yz & z^{2} & x & y & z & 1 \end{array}\right]}^{T}\;\alpha ={\left[\begin{array}{ccccc} a_{1} & a_{2} & a_{3} & \ldots & a_{10} \end{array}\right]}^{T}.$$

The measurement data of the ellipsoid fitting are to solve the coefficient of the ellipsoid. It is to meet all the sum of the squares of the algebraic distance measurement data to the ellipsoid minimum as $\underset{\alpha }{\arg\min}\left(E\right)$ .

$$E=\sum_{i=1}^{N}F{\left(\alpha ,x_{i}\right)}^{2}={\left\|\begin{array}{cc} D & \alpha \end{array}\right\|}^{2}.$$ (7)

$D={\left[\begin{array}{ccccc} X_{1} & X_{2} & X_{3} & \ldots & X_{N} \end{array}\right]}^{T}$ , which is an N×6 matrix.

To guarantee that the quadric surface is an ellipsoid, to satisfy the following constraints,

$$\det\left(W\right)>0\Leftrightarrow 4a_{1}a_{3}-a_{2}^{2}>0,$$ (8)

$$\left(a_{1}+a_{3}\right)\det\left(A\right)>0.$$ (9)

$W=\left[\begin{array}{cc} a_{1} & a_{2}/2\\ a_{2}/2 & a_{3} \end{array}\right]$ and $A=\left[\begin{array}{ccc} a_{1} & a_{2}/2 & a_{4}/2\\ a_{2}/2 & a_{3} & a_{5}/2\\ a_{4}/2 & a_{5}/2 & a_{6} \end{array}\right]$ . As the free parameters, α can be of suitable magnification, making Eq. 8 satisfies $4a_{1}a_{3}-a_{2}^{2}=1$ .

$$\alpha^{T}C\alpha =1.$$ (10)

The solution satisfied the constraint conditions of the matrix Eq. 7, using the Lagrange multiplier method:

$$\{\begin{array}{c} D^{T}D\alpha =\lambda C\alpha ,\\ \alpha^{T}C\alpha =1,\\ \left({\text{a}}_{1}+a_{2}\right)\det\left(A\right)>0. \end{array}$$ (11)

By solving Eq. 11, the coefficient α of the ellipsoid for the least positive characteristics of the corresponding eigenvectors will be obtained.

According to the special structure of the matrix, through the matrix decomposition, we can overcome the defects of the constraint matrix which is singular, and simplify the feature vector to solve the following equations.

First, $D=\left[D_{1}\;D_{2}\right]$ and $D_{1}={\left[\begin{array}{ccc} x_{1}^{2} & x_{1}y_{1} & y_{1}^{2}\\ \vdots & \vdots & \vdots \\ x_{i}^{2} & x_{i}y_{i} & y_{i}^{2}\\ \vdots & \vdots & \vdots \\ x_{N}^{2} & x_{N}y_{N} & y_{N}^{2} \end{array}\right]}_{N\times 3}$ $D_{2}={\left[\begin{array}{ccccccc} x_{1}z_{1} & y_{1}z_{1} & z_{1}^{2} & x_{1} & y_{1} & z_{1} & 1\\ \vdots & \vdots & \vdots & \vdots & \vdots & \vdots & \vdots \\ x_{i}z_{i} & y_{i}z_{i} & z_{i}^{2} & x_{i} & y_{i} & z_{i} & 1\\ \vdots & \vdots & \vdots & \vdots & \vdots & \vdots & \vdots \\ x_{N}z_{N} & y_{N}z_{N} & z_{N}^{2} & x_{N} & y_{N} & z_{N} & 1 \end{array}\right]}_{N\times 7}$ ,then $S=D^{T}D=\left[\begin{array}{cc} S_{1} & S_{2}\\ S_{3} & S_{4} \end{array}\right]$ , and $S_{1}=D_{1}^{T}D_{1}$ , $S_{2}=D_{1}^{T}D_{2}$ , $S_{3}=D_{2}^{T}D_{1}=S_{2}^{T}$ , and $S_{4}=D_{2}^{T}D_{2}$ .

We can get the constrain matrix $C=\left[\begin{array}{cc} C_{1} & C_{2}\\ C_{3} & C_{4} \end{array}\right]$ and $C_{1}=\left[\begin{array}{ccc} 0 & 0 & 2\\ 0 & -1 & 0\\ 2 & 0 & 0 \end{array}\right]$ , $C_{2}={\left[0\right]}_{3\times 7}$ , $C_{3}={\left[0\right]}_{7\times 3}$ , $C_{4}={\left[0\right]}_{7\times 7}$ , making $\alpha =\left[\begin{array}{c} \alpha_{1}\\ \alpha_{2} \end{array}\right]$ ,and $\alpha_{1}={\left[a_{1}\;a_{2}\;a_{3}\right]}^{T}$ , $\alpha_{2}=\left[a_{4}\;a_{5}\;a_{6}\;a_{7}\;a_{8}\;a_{9}\;a_{10}\right].$

Substituting the aforementioned matrix decomposition into (11), we can get

$$S_{1}\alpha_{1}+S_{2}\alpha_{2}=\lambda C_{1}\alpha_{1},$$ (12)

$$S_{2}^{T}\alpha_{1}+S_{4}\alpha_{2}=0.$$ (13)

When the sampling data are not in the same plane, $S_{4}$ is a singular matrix [12], finishing available:

$$C_{1}^{-1}\left(S_{1}-S_{2}S_{4}^{-1}S_{2}^{T}\right)\alpha_{1}=\lambda \alpha_{1},$$ (14)

$$\alpha_{2}=-S_{4}^{-1}S_{2}^{T}\alpha_{1}.$$ (15)

Then, Eq. 10 can be changed into

$$\alpha_{1}^{T}C_{1}\alpha_{1}=1.$$ (16)

The aforementioned matrix decomposition combines Eq. 11 with Eqs 14–16 solutions that get the minimum corresponding eigenvectors, characteristic root, and plug in Eq. 9. This formula (11) solving the 10-day feature vector into the formula (14) solution of the three-dimensional feature vector to decrease the amount of calculation for about a third of the original, and at the same time, using the improved algorithm on accuracy is consistent with the original algorithm.

According to Eqs 5, 6, matrices A , b can be obtained, and because of Eq. 10 for amplification coefficient α , the matrix is relative. As the absolute value of A magnification by Eqs 5, 6, the corresponding relation can be obtained:

$$k=a_{10}/\left(b^{T}Ab-H^{2}\right).$$ (17)

Calculated according to Eq. 17, matrices A , b , and by Eq. 5 work out the corresponding relationship between the error compensation coefficient matrix $K_{c}$ and $B_{c}$ , and complete the error compensation.

In order to validate the aforementioned algorithm, simulation software is used. Assuming that the magnetic sensor location in the uniform magnetic field with a magnetic field strength of 0.52 G, we divide the ideal output $h_{t}$ spherical area into N regions, and each segmented region is the random selection of measuring point data (Patel and Holtzman, 1994; Jiang et al., 2019a; Zhao et al., 2022).

**FIGURE 2:**
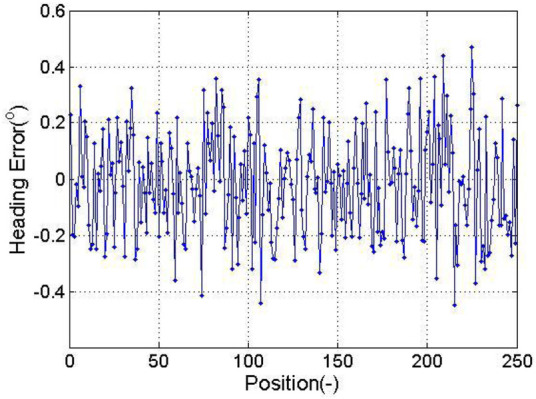
Heading error of simulation.

To test algorithm’s calculation accuracy, a given magnetic sensor attitude as a benchmark and the computed error compensation of the magnetic sensor course are used. Record of location, respectively, sets the pitching angle and tilt angle, pitching angle and tilt angle, pitching angle and tilt angle, and pitching ngle and tilt angle for the round, and each group of uniform records 36 points. Calculation of the yaw angle error is shown in Figure 2.

## Acquisition of a Motion Track of the Vibrating Screen

### Acquisition of the Vibration Signal

**FIGURE 3:**
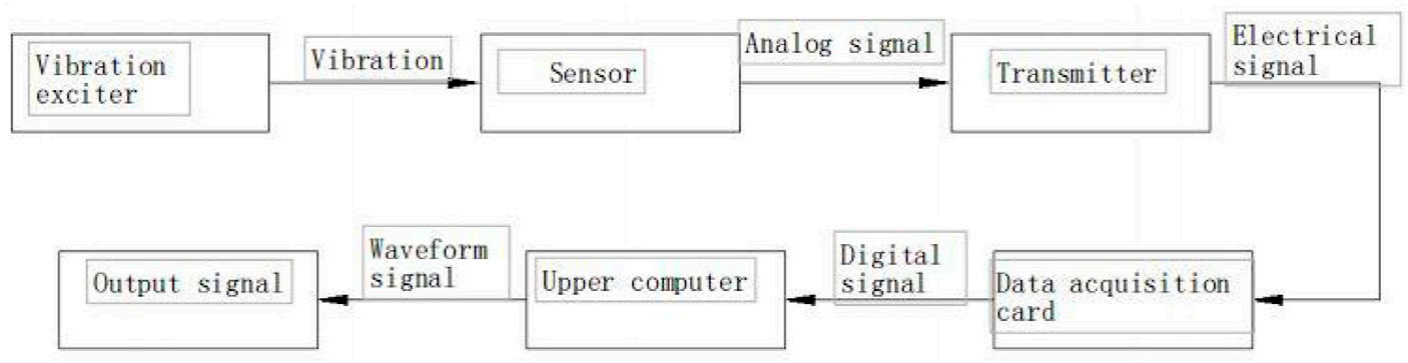
Working principle.

The vibrating screen studied in this work is an elliptical vibrating screen. The motion track of the elliptical vibrating screen is determined by the relationship between the frequency, amplitude, and phase angle of the vibrating screen during vibration. The working principle of the elliptical vibrating screen monitoring system is shown in Figure 3.

The vibration frequency of the vibrating screen is between 0 and 24 hz and the amplitude is between 0 and 5 mm. After debugging the relevant parameters, the computer, monitoring hardware, and vibrating screen must be energized. Using four steel plate foot sleeves, four gravity sensors are put into the four steel foot sleeves, then four feet of the vibrating screen are put on four sensors. The sensor is under pressure. At this time, the line with the zero waveform starts to fluctuate until the force on the sensor is stable and the wave shape gradually becomes a stable horizontal line, and then the power is turned on. After the power is turned on, the vibrating screen starts to vibrate. With the vibration of the vibrating screen, the gravity sensor will be impacted to varying degrees and then transmitted to the data acquisition card through the transmitter. A continuously changing waveform will be displayed on the waveform display interface of the computer software. The change of the waveform will not only change with different motion frequencies of the vibrating screen but the amplitude of the frequency will also be different with the different impact forces of the vibrating screen. Moreover, this change is generally not periodic, but random (this experiment is carried out when one sensor works, that is, the sensor is placed under one corner of the vibrating screen, and the other three corners are leveled with other articles) (Kohno et al., 1983; Verdu, 1986; Jiang et al., 2019b).

### Denoising and Amplification of the Vibration Signal

**FIGURE 4:**
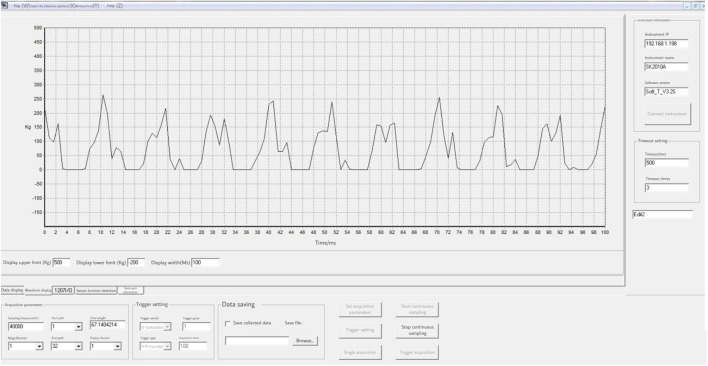
Vibration signal distortion.

The vibration signal of the vibrating screen is transmitted to the transmitter by the sensor, then the vibration signal (vibration signal is a non-electric signal) transmitted by the sensor is transformed into a voltage signal under the action of the transmitter, and then the transformed voltage signal is amplified to facilitate the measurement and control of the following signals. Because the vibration signal is weak during detection, it is easily disturbed by the surrounding environment and media during monitoring (Wang, 1998; Woodward and Vucetic, 1998; Jiang et al., 2021b). After being disturbed, the detected signal will produce distortion, as shown in Figure 4, which is the sinusoidal image monitored by vibration signal distortion.

Most of the reasons for the waveform distortion of the motion track of the vibrating screen are because the accuracy of the acceleration value measured by the acceleration sensor is not high when the vibrating screen is vibrating, so the waveform presented in the upper computer is distorted.

Denoising: for this phenomenon, the general method is to use the fast Fourier transform (FFT) method to solve the problem of inaccurate signals detected by the acceleration sensor. FFT has a fast and prominent amplitude-frequency analysis ability to timely determine the frequency, phase, and amplitude of the waveform, to modify the parameters of the vibrating screen during vibration and ensure the smooth operation of the vibrating screen (Madhow, 1998; Li et al., 2017; Liu et al., 2022a).

Amplification: the internal structure of the transmitter comprises many amplifiers. The vibrator converts the analog signal sent by the sensor into a voltage signal. The amplifier inside the transmitter will amplify the weak voltage signal received. Even if the noise is large, the voltage signal is submerged in the noise (Duel-Hallen et al., 1995; Liu X et al., 2022; Wu et al., 2022). After amplification by the amplifier, the voltage signal is easier to detect.

### Calculation of the Vibration Signal

The motion track of the vibrating screen is determined by the relationship between the frequency, amplitude, and phase angle of the vibration. The vibrating screen detects the electrical signal of the acceleration sensor through vibration, expresses the electrical signal in the form of waves through the computer software, and then expresses it in the vibration image of the *X*, *y,* and *Z* axes of the vibrating screen. The motion trajectories of any two axes of three axes are fitted and simulated (Klein et al., 1996; Liu et al., 2022b; Yun et al., 2022).

The following is the relationship expression of amplitude, frequency, and phase angle on three axes:

Time is expressed in t

$$\text{X}\;\text{axis:}\;\text{X}={\text{A}}_{1}\text{sin}\left(2\pi ft+\varphi_{1}\right),$$ (18)

$$\text{Y}\;\text{axis:}\;\text{Y}=A_{2}\text{sin}\left(2\pi ft+\varphi_{2}\right),$$ (19)

$$\text{Z}\;\text{axis:}\;\text{Z}=A_{3}sin2\pi ft,$$ (20)

where A1, A2, and A3 represent the amplitudes in the *X*, *y,* and *Z* axes, respectively;

$\varphi_{1},\varphi_{2}$ represent the phase angles on the *X* and *Y* axes;

F represents the frequency; and

T represents time.

Because the research object of this study is the elliptical vibrating screen, it is enough to fit the moving track of the vibrating screen as an ellipse with VC + + on the XY axis of the plane coordinate, that is, the ellipse is the moving track of the vibrating screen.

## Testing of the Data Acquisition Module

The data acquisition module of the monitoring system is combined with TP410 to realize the simultaneous acquisition of multiple modules, USB2.0 communication output, upper computer software display, unified display, acquisition, recording, control, and analysis. It can collect and analyze a variety of sensor signals, including pressure, flow, liquid level, temperature, displacement sensors, etc. An independent configuration channel is set, and the effective resolution is 16 bits. It can realize a communication distance of 500 m without shielding, and the communication frequency band is 2.5 hz. At the same time, it can adapt to various working environments such as −10°C ∼ + 70°C.

**FIGURE 5:**
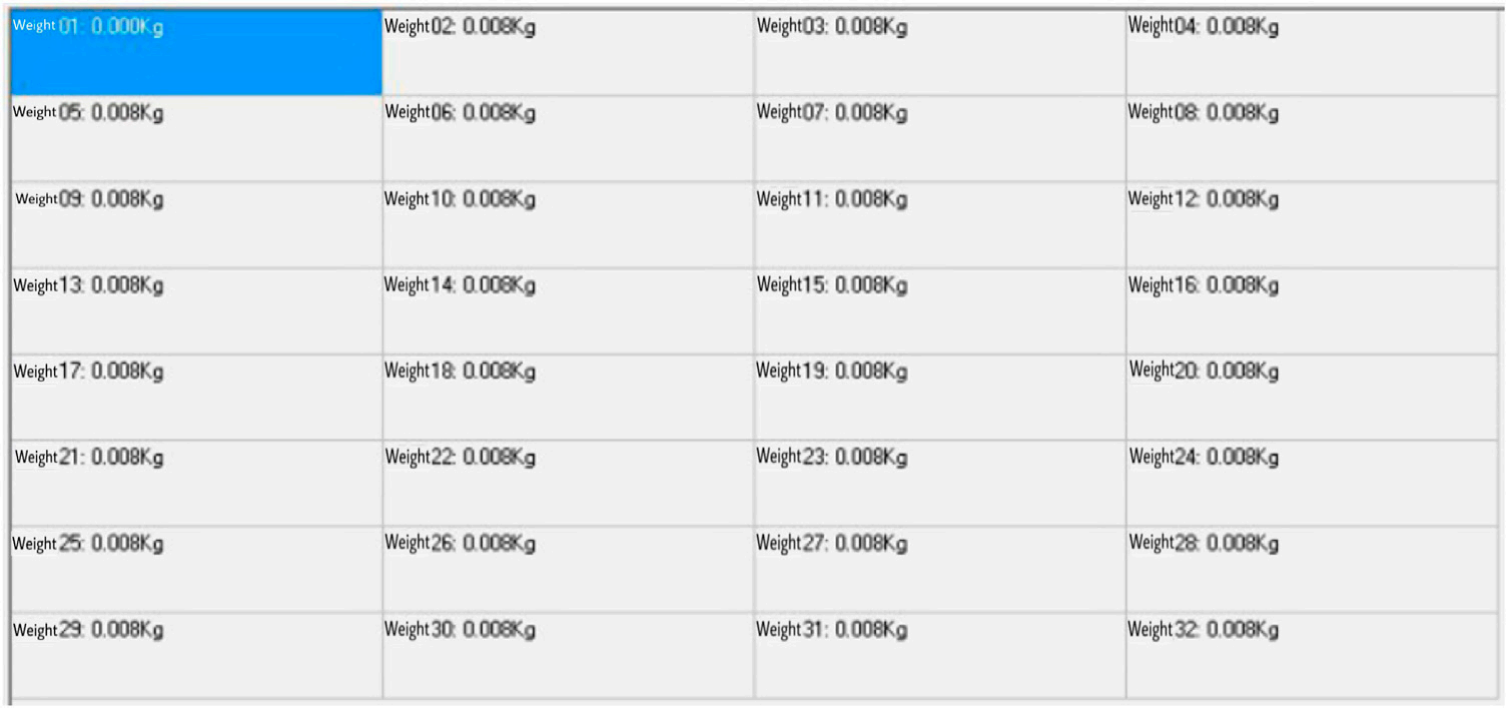
Data display of the acquisition channel.

The monitoring system includes a total of 32 data acquisition channels. Each channel collects information independently of each other. Finally, it is summarized in the display window, and the auxiliary waveform accurately displays the operation status of the equipment. During calibration, each channel is tested one by one, the pressure sensor is placed under the hydraulic press, a fixed load and a dynamic load, respectively, are applied, the signal is collected and fed back to the signal amplifier through the sensor for signal processing, the information is collected, processed, and transmitted through the data acquisition card, and the test results are displayed through the monitoring platform. Each channel is tested one by one and the experiment is repeated until all channels are tested accurately. The data collected by the acquisition channel are shown in Figure 5.

**FIGURE 6:**
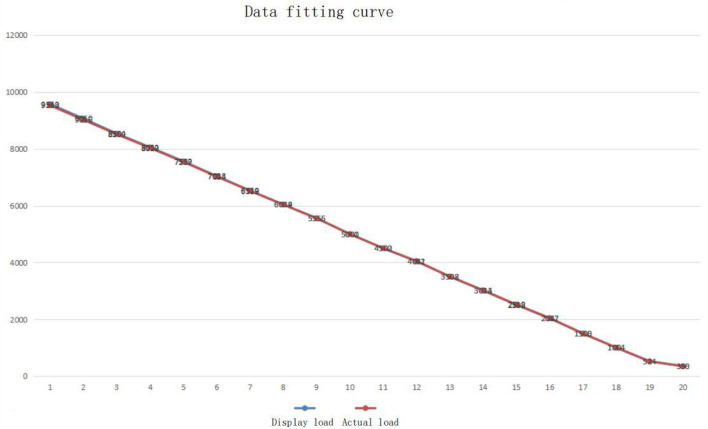
Fitting image of collected data and applied load.

The collected data are organized into a table, the actual load applied by the hydraulic press is analyzed, the sensor measurement structure is compared, and the test load curve and the collected measurement curve are drawn in the same table for fitting. The fitting curve between the actual load and the collected data can be observed, and the two lines almost completely coincide, indicating that the data acquisition module operates normally. The data acquisition and fitting images are shown in Table 3 and Figure 6.

**TABLE 3:** Comparison of data sets.

Sensor 1 (channel I)						Sensor 2 (channel 2)					
Positive testing			Reverse test			Positive testing			Reverse test		
Display load	Actual load	Error	Display load	Actual load	Error	Display load	Actual load	Error	Display load	Actual load	Error
119	110	9	9586	9510	76	505	502	3	9075	9010	65
517	512	5	9110	9039	71	1015	1010	5	8521	8467	57
1008	1007	I	8568	8506	62	1532	1526	6	8062	8011	51
1503	1516	−13	8073	8015	58	2045	2045	0	7574	7530	44
2012	2012	0	7568	7520	48	2520	2518	2	7040	7007	33
2502	2503	−1	7085	7046	39	3010	3010	0	6500	6572	−72
3012	3007	5	6548	6520	28	3502	3506	−4	6040	6025	15
3517	3510	7	6026	6005	21	4010	4054	−14	5503	5500	3
4020	4014	6	5526	5513	13	4500	4503	−3	5018	5019	−1
4518	4512	6	5012	5006	6	5018	5014	4	4521	4529	−8
5017	5008	9	4527	4529	−2	5520	5514	6	4020	4035	−15
5524	5500	24	4013	4016	−3	6030	6012	18	3495	$503	−8
6031	6005	26	3503	3508	−5	6532	6508	21	3020	3030	−10
6530	6500	30	3010	3015	−5	7050	7017	33	2507	2514	−7
7010	7000	40	2509	2514	−5	7540	7491	49	1998	2000	−2
7553	7506	47	2032	2041	−9	8085	8030	55	1522	1522	0
8070	8009	61	1518	1518	0	8580	8525	55	1020	1020	0
8616	8550	66	1020	1010	10	9076	9018	58	525	519	6
9093	9021	72	526	510	16				396	390	6
9553	9470	83	315	303	12				296	290	6
			207	196	II						

## Summary

The vibrating screen will be subjected to a static load when leaving the factory. The verification of the dynamic load and the research of its motion trajectory have important application value. The research object of this study is a 20 t elliptical vibrating screen. Through the construction of a hardware and software system, and a real-time system monitoring and motion trajectory fitting, the following conclusions can be drawn:

1) This study takes the 20 t vibrating screen as the research object. Through the construction of a software and hardware system and the simulation and simulation software written by VC + +, the real-time monitoring of the vibrating screen is realized.
2) The vibrating screen will emit strong vibration during operation, which will have an immeasurable impact on the surrounding environment and buildings. Therefore, the vibrating screen must be monitored by a real-time monitoring system when leaving the factory. Spherical error compensation improves trajectory accuracy.
3) The designed hardware and software have certain reliability for whether there are dangerous factors during the operation of the vibrating screen.

## Data Availability

The original contributions presented in the study are included in the article/Supplementary Material; further inquiries can be directed to the corresponding author.
